# The same, only different: Smartphone‐based dietary Ecological Momentary Assessment tools vary in complexity, usability and active information processing

**DOI:** 10.1111/bjhp.70057

**Published:** 2026-01-28

**Authors:** Anila Allmeta, Stephen Sutton, Laura M. König

**Affiliations:** ^1^ Faculty of Life Sciences: Food, Nutrition and Health University of Bayreuth Kulmbach Germany; ^2^ Department of Public Health and Primary Care University of Cambridge Cambridge UK; ^3^ Faculty of Psychology University of Vienna Vienna Austria

**Keywords:** ambulatory assessment, digital dietary assessment, food intake, mHealth, mobile apps

## Abstract

**Objectives:**

Ecological Momentary Assessment (EMA) is popular for assessing dietary intake in real life and real time. Available tools differ substantially in the type and number of implemented features, including features to assess what and how much was consumed. These features require qualitatively different input that might have a differential impact on the participants' cognitions and behaviours while taking part in the study. This study aimed to test whether more complex dietary assessment tools, indicated by the type and number of assessment features, induce more active information processing (AIP).

**Design:**

Preregistered online between‐subjects experimental study.

**Methods:**

A total of 373 participants (65.4% female; mean age 30.4 years) were randomly allocated to view one of eight EMA protocol mock‐ups, each describing a food tracking process verbally and using screenshots. Afterwards, they rated the protocol in terms of its complexity (manipulation check), AIP and its potential impact on eating‐related cognitions, intentions and eating behaviour change.

**Results:**

The eight EMA protocols differed in perceived complexity, that is protocols with more tracking features were perceived as more complex compared to those with fewer tracking features. EMA protocols that were perceived to be more complex were also perceived to induce more AIP. However, there were no differences in perceived impact on eating‐related cognitions, intentions and behaviour.

**Conclusions:**

Differences in complexity and usability may influence compliance and study results. Researchers thus need to carefully select the appropriate EMA protocol for their study to balance the need for collected information with the need for high compliance.

**Statement of Contribution**

**What is already known?**

- Smartphone‐based Ecological Momentary Assessment (EMA) is increasingly popular for assessing diet.
- Taking part in these studies may influence study participants' eating behaviour.

**What does this study add?**

- Dietary EMA tools vary in complexity, depending on the number and type of recording features.
- These differences are unlikely to differentially influence eating‐related cognitions, intentions and behaviours.
- Usability considerations may influence the choice of the most appropriate dietary EMA tool.

## INTRODUCTION

Smartphone‐based dietary assessment via Ecological Momentary Assessment (EMA) (Shiffman et al., 2008) is becoming increasingly popular in health psychology and related fields to assess a range of health behaviours (Ottenstein & Werner, 2022; Perski et al., 2022; Wrzus & Neubauer, 2023), including dietary behaviours (König, van Emmenis, et al., 2022). It enables food intake in daily life to be assessed without recall bias (Sharp & Allman‐Farinelli, 2014), in relation to various determinants (Perski et al., 2022) and across time, which may lead to more accurate measurement and more robust findings and subsequently may advance psychological theories (Scholz, 2019).

A multitude of smartphone‐based dietary assessment tools have been used in research that differ substantially in the type and number of implemented features (König, van Emmenis, et al., 2022). For instance, some tools require participants to take photos (König & Renner, 2018), some ask participants to search for individual food items in a database (Ambrosini et al., 2018) or through classification systems (Biel et al., 2018; Elliston et al., 2017) and indicate portion sizes (Béjar et al., 2018; Jung et al., 2020), and others ask for a free‐text description of the food intake (König & Renner, 2018, 2019).

These features require qualitatively different input. It could be hypothesized that they also exert differential impact on participants' cognitions and behaviours while taking part in the study. Indeed, prior research both in the context of surveys and intensive longitudinal assessments suggests that study participants' behaviours, cognitions or emotions may change due to study participation, a phenomenon known as measurement reactivity (French & Sutton, 2010). Effects are often small, but may still be meaningful, given that effects of behavioural interventions also typically report small to medium effect sizes (König, Allmeta, et al., 2022; Maher et al., 2024; Miles et al., 2020). Studies specifically testing reactivity to the measurement of dietary intake are sparse. Research in survey studies has produced highly heterogeneous findings so far, and according to a recent systematic review, (König, Allmeta et al., 2022) the issue has not been addressed at all in intensive longitudinal studies on diet. The literature also speculates about the potential role of involving participants actively in the recording process. For example, one study found stronger reactivity effects when participants were explicitly asked to copy the step count from their step counter into a paper diary compared to when they were not (Clemes & Parker, 2009).

When recording dietary intake, the various tools also require different interactions with the recording tools, which may induce different levels of active processing of information such as sensory input while recording. In other contexts, more active processing of information is related to better understanding of information and performance (Natter & Berry, 2005). It could thus be assumed that more active information processing (AIP) in dietary assessment leads to more pronounced changes in eating behaviour (see also Barta et al., 2012). For example, selecting individual foods from a database and adding portion sizes requires study participants to provide detailed information on their meals, which demands active reflection. Taking a picture of the meal, however, can be completed in seconds and might require less active reflection. Thus, depending on which assessment tool is used to record food intake, AIP and reflection on the food choices might vary substantially. In the present study, AIP is defined as the active reflection on the information that the participants have to provide while recording their food intake using different recording features (i.e., photo‐based, food database, detailed description). If these differences indeed translate into behaviour, researchers would need to be careful when comparing data from studies that used dietary assessment tools with different features.

The present study provides a first test of these assumptions. Specifically, the study aimed to test whether more complex dietary assessment tools, where complexity is indicated by the type and number of assessment features, induce more active information processing. We hypothesized that assessment tools that, for instance, require participants to describe individual components by searching in a food database and input serving sizes, are perceived (i.e., rated by the participants) to be more complex (H1), to induce more AIP (H2) and to subsequently produce greater changes in eating‐related cognitions, intentions and behaviour (H3) (c.f., French & Sutton, 2010) than less complex tools (e.g., taking a photo only). Furthermore, the study aimed to explore the usability of the different dietary assessment tools (exploratory research question; E1), which is more often reported for assessment or intervention apps (König, van Emmenis et al., 2022) and linked to the complexity of tools; and to test whether previous experience with nutrition apps (E2) and the level of affinity to technology (ATI) (E3), that is an individual's tendency to actively engage with technology (Franke et al., 2019), influenced participants' perceptions on active information processing, eating‐related cognitions, intentions and behaviours. Taking these individual characteristics into account is important since health behaviour tracking through EMA has become popular in recent years (König et al., 2018; Pew Research Center, 2020), and experience and ATI may influence engagement with the tracking device (Seifert et al., 2017) and thus moderate the postulated effects.

## METHODS

This study was preregistered on the Open Science Framework (OSF; https://osf.io/5gtqd), and all study materials and data are available from the OSF (https://osf.io/4htx7/). The study protocol was approved by the Ethics Committee of the University of Bayreuth.

### Sample

A power analysis in GPower 3.1 (Faul et al., 2009) indicated a sample size of *N* = 368 for an alpha level of .05, and a small to medium effect size (Cohen's *d* = .4 or $\eta_{p}^{2}$ = .06) (Cohen, 1992; Lakens, 2013) using a one‐way between‐subjects ANOVA with eight groups for 80% power. Inclusion criteria were being at least 18 years old, having a good command of the English language and having access to a smartphone/computer/tablet for the duration of the study.

A total of 373 participants completed the questionnaire; they were recruited through social media (i.e., the official Instagram page of the faculty where the study was conducted; on the authors' professional and personal social media profiles on Instagram and Twitter/ X, special interest groups pages within the psychology community) and word of mouth in spring and summer 2021. The mean age of the participants was 30.4 years (*SD* = 9.8) with 65.4% women, 34.1% men and .5% indicating another gender. The majority of the participants held a university degree (42.1% Bachelor's degree, 24.1% Master's degree). Regarding previous experience with nutrition apps, 56.3% stated not to have any experience, whereas 43.7% (*n* = 163) reported having some experience (Table 1). The most frequently used nutrition apps were My Fitness Pal (39.2%), Yazio (11.9%), Fitbit (10.8%) and Lifesum (9.7%).

**TABLE 1:** Participants' prior experience with nutrition apps.

Stage	*N*	%
1: I have never thought about using an app for that	101	27.1
2: I have thought about using an app for that, but so far, I did not do it	73	19.6
3: I have thought about using an app for that, but it is not necessary for me to do it	36	9.7
4: I am currently using an app for that and intend to continue to use it	38	10.2
5: I have used an app for that, but I do not use it anymore	125	33.5

### Study design and procedure

An experimental online study with one between‐subject factor (*EMA protocol*) was conducted. All participants provided informed consent before participating in the study. Participation was voluntary, anonymous and participants did not receive any compensation or feedback. The online study was conducted using the survey platform Tivian Unipark and lasted for approximately 15 minutes. First, participants provided demographic information; then they were randomly allocated by the survey platform to view one of the eight EMA protocols, each describing a food tracking process reflecting a typical nutrition app in the form of mock‐ups. The reporting process included a series of screenshots of the hypothetical app and a written description of each step. Afterwards, they rated the hypothetical assessment tool in terms of its complexity (manipulation check), active information processing and its potential impact on eating‐related cognitions, intentions and eating behaviour change. Then, participants completed the short form of the User Experience Questionnaire (Schrepp et al., 2017). Finally, they provided information on their experience with nutrition apps and completed the Affinity to Technology (Franke et al., 2019) short scale (Wessel et al., 2019).

### Materials and measures

#### EMA protocols

Eight draft protocols were created in the form of mock‐ups of typical nutritional apps that were identified based on the systematic review by König, van Emmenis, et al. (2022). The mock‐ups included screenshots of the assessment tool to show how the tool would look, as well as written descriptions of the recording process to explain how the tool should be used. The design of the mock‐ups (i.e., colour scheme, icons, font) was identical across the eight mock‐ups for consistency. In the study, participants were asked to read the description, look at the screenshots and take their time to imagine that they would be using the nutrition app to record their food intake (breakfast, lunch, dinner, snacks and drinks) several times per day for several consecutive days.

**FIGURE 1:**
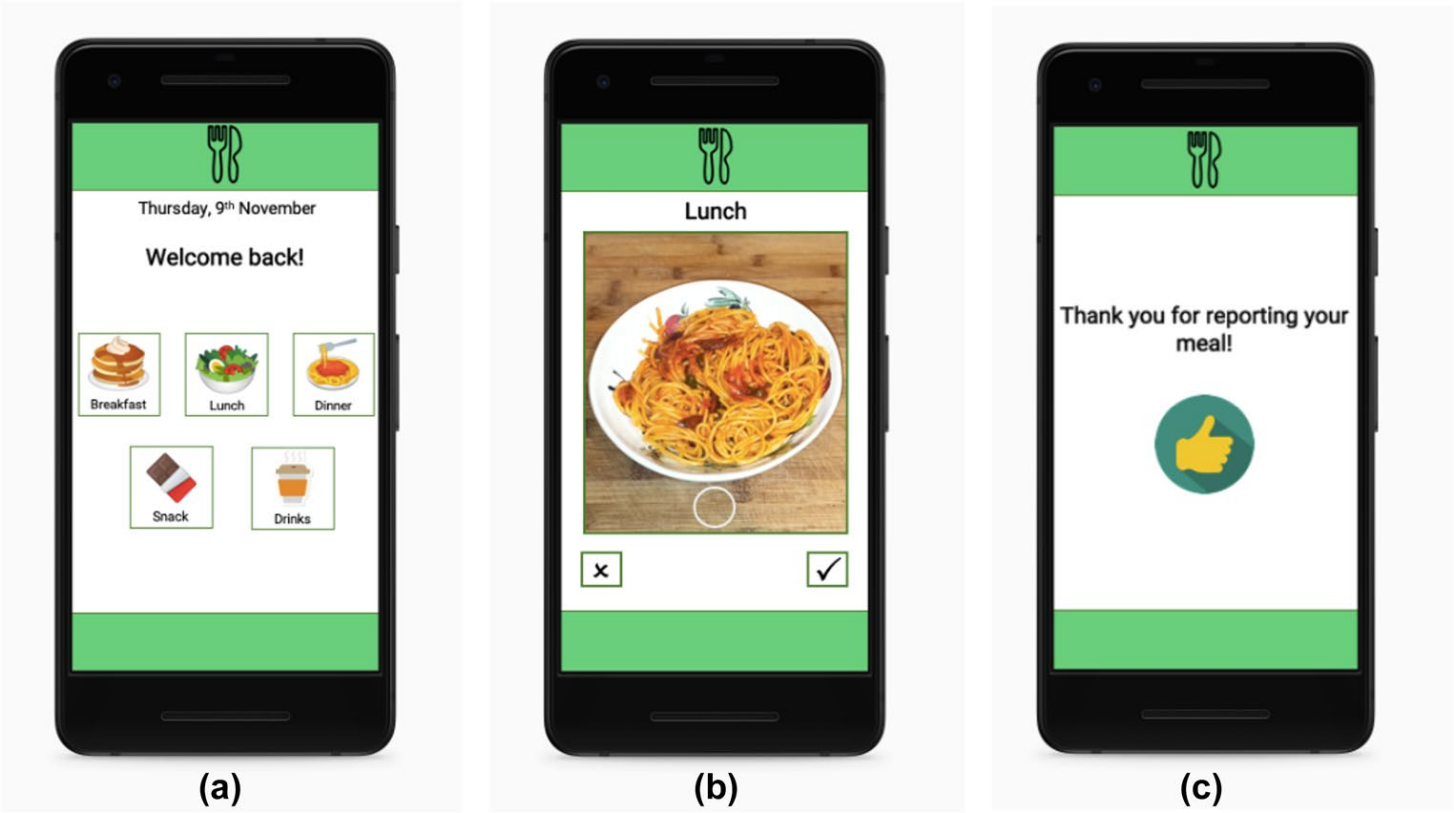
EMA protocol 3, depicting the simplest recording process only containing the photo feature. This version of the app contained three screenshots, depicting (a) the meal type selection, (b) taking a photo of the meal, (c) a thank you screen confirming the recording.

**FIGURE 2:**
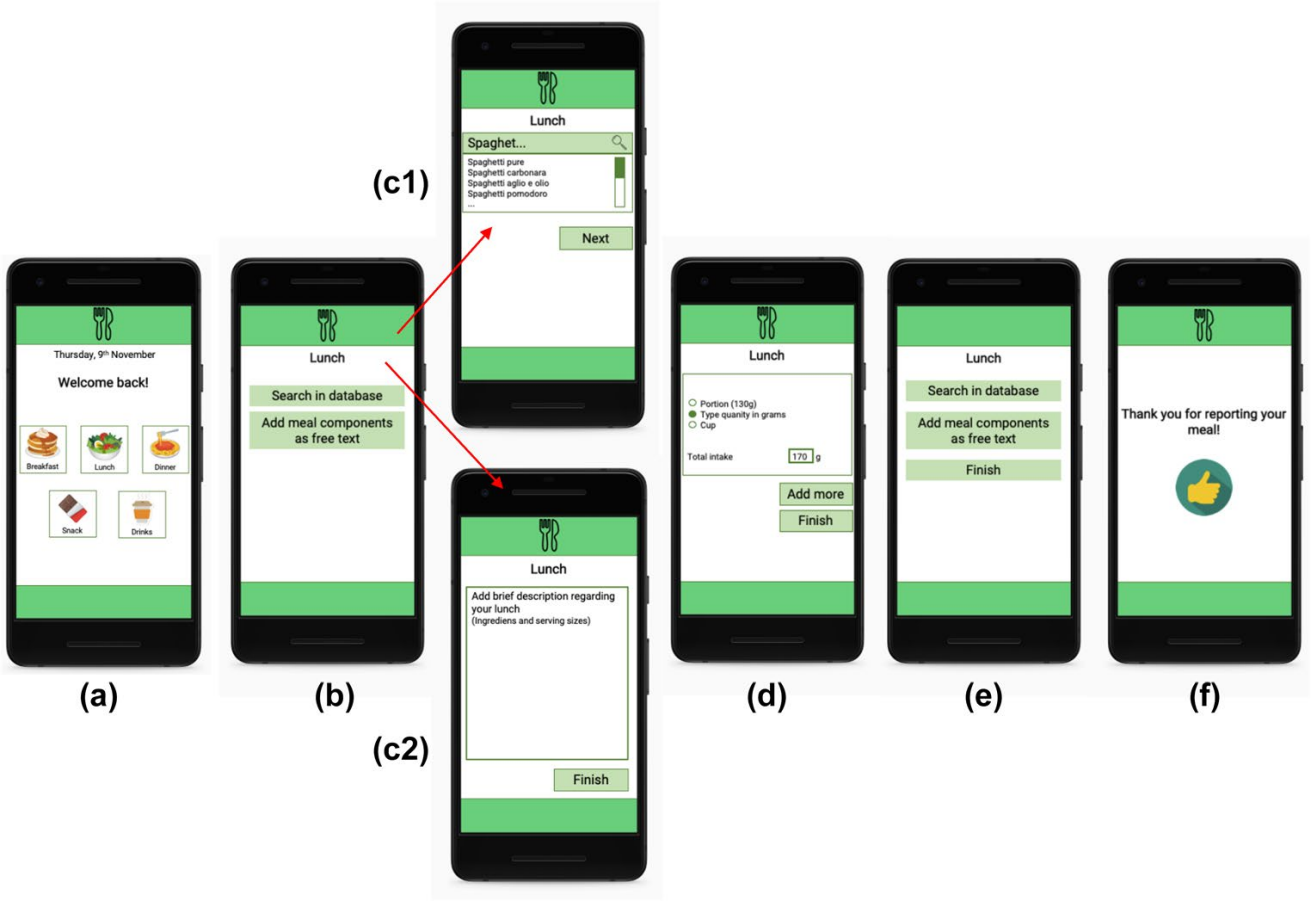
EMA protocol 7, containing four recording features. This version of the app contained seven screenshots, depicting (a) the meal type selection, (b) selecting whether to search in a database or add meal components as free text, (c‐1) the database, (c‐2) the open text field, (d) the portion size estimation, (e) adding another component or finish, (f) a thank you screen confirming the recording.

All mock‐ups included a first screenshot in which the user would hypothetically choose to record a meal (breakfast, lunch, dinner), a snack or a drink. The rest of the screenshots that followed differed for the eight protocols; their components were based on the five core features of nutrition apps identified in König, van Emmenis, et al. (2022): photo‐based assessment, food database, classification system, free‐text description, assessment of serving or portion size. The combination of features in the different mock‐ups is presented in Table 2. The eight mock‐ups were assumed to differ in complexity. Initially, we conceptualized complexity via information load, for which the number of screenshots and the number of words used to describe the recording process were used as pre‐defined indicators (see Table 2). Depending on the mock‐up the participant was shown (and thus the tracking feature combinations), they would hypothetically either take a photo of their meal, describe in detail the ingredients of the meal, search the meal in a food database, categorize the food into food categories (i.e., vegetables and fruits, starchy food), or report the serving size (i.e., cups, portions or grams). Finally, in all protocols, the mock‐up included the same last screenshot confirming the recording. Figure 1 illustrates EMA protocol 3, in which only the photo recording tracking feature was used, while Figure 2 illustrates EMA protocol 7, in which four features (photo recording, food database, detailed description, serving size) were combined. All screenshots are available from the project's OSF page.

**TABLE 2:** Description of the EMA protocol mock‐ups used in the study, including the tracking features, number of screenshots and length of the description.

EMA protocol no.	Tracking features	Number of screenshots	Words
1	Detailed description	3	91
2	Classification system	3	95
3	Photo recording	3	112
4	Classification system + Serving size	4	134
5	Food Database + Serving size	4	152
6	Photo recording + Detailed description	4	159
7	Food Database + Detailed description + Serving size	7	318
8	Photo recording + Food Database + Detailed description + Serving size	8	383

#### Perceived complexity (manipulation check)

Participants were asked to rate the complexity of the EMA protocol they were shown on six items on a 5‐point Likert scale (1‐strongly disagree to 5‐strongly agree). For instance, they rated whether recording meals and snacks with the app was detailed and whether it required a lot of input. In all the following sections of the paper, the complexity of the EMA protocols refers to these ratings.

#### Active information processing

AIP was assessed with 7 items using the same 5‐point Likert scale (1‐strongly disagree to 5‐strongly agree). Items were developed by the study team, which comprised experts in the study of the psychology of eating, including underlying cognitive processes. For instance, the participants rated whether recording a meal/snack with the app required them to think about what they were about to eat or required them to know exactly how much they were about to eat.

#### Eating‐related cognition, intentions and behaviours

Eating‐related cognitions were assessed with 6 items. For example, participants indicated whether using the study app made them think about the healthiness of the meal or become aware of how much they ate. Intentions to change behaviour were assessed with 5 items. For instance, participants rated whether using the study app made them want to eat less or want to eat fewer snacks. Anticipated changes in eating behaviour were assessed through 5 items. Amongst others, participants rated whether using the study app would make them eat less or eat healthier. The same 5‐point Likert scale was used in these three measures (1‐strongly disagree to 5‐strongly agree). Although this particular measure was self‐developed to ensure maximum fit for the research question as well as brevity, the items were inspired by items used in the eating behaviour literature, which frequently assessed eating‐related cognitions (e.g., regarding when, where and how participants consumed food; Wansink & Sobal, 2006), intentions (Reichenberger et al., 2019) and behaviours (e.g., meal skipping, Pendergast et al., 2016).

### User experience questionnaire (UEQ)

The short form of the user experience questionnaire (Schrepp et al., 2017) was used to assess the usability of the EMA protocols. The short form of the questionnaire (8 items) was developed based on the original long version of the UEQ (26 items) and has been translated into 19 different languages (see Schrepp et al., 2017 for development, validation and reliability). Participants were presented with 8 semantic differentials (4 pragmatic and 4 hedonic quality items) such as complicated‐easy; confusing‐clear. They were asked to indicate their response using a 7‐point Likert scale from 1 = negative connotation to 7 = positive connotation. An overall mean of the scale was calculated; higher values represent higher usability of the EMA protocols.

### Experience with using nutrition app

Participants' previous experience in using nutrition apps was assessed using a stage model approach as presented by König et al. (2018). When they indicated that they had used an app before or were currently using an app to track their food intake, they were asked to state the app's name in an open text field.

### Affinity to technology (ATI)

ATI is a scale used to measure an individual's general comfort and willingness to engage with technology. Participants indicated their degree of interaction with technical systems (i.e., apps, software, mobile phones, PC) by indicating agreement with the four statements in the ATI (Wessel et al., 2019) scale short form. The reliability and validity of the scale have been confirmed (Franke et al., 2019; Heilala et al., 2022; Lezhnina & Kismihók, 2020), and the scale has been translated into different languages (University of Lübeck, 2025). Due to a programming mistake, responses were assessed on a 5‐point instead of a 6‐point Likert scale (1 = completely disagree; 5 = completely agree). The responses for the two negatively worded items (items 3 and 4) have been reversed before calculating the mean of the items; higher values represent higher affinity to technology.

### Statistical analysis

Analyses were conducted using IBM SPSS Statistic software version 29.0.0.0. Missing values were deleted case‐wise. Missing values ranged from 0% for active information processing, eating‐related cognition, intention and behaviour to 2.1% for perceived complexity, 2.4% for ATI and 3.2% for previous experience with nutrition apps. The main research questions (H1–3) were investigated by conducting one‐way between‐subject ANOVAs using the eight EMA protocols as the independent variable and the respective sub‐scales for complexity, active information processing, eating‐related cognitions, intentions and behaviour as the dependent variables.

The first exploratory question (E1) was tested using a one‐way between‐subjects ANOVA, with the eight EMA protocols as the independent variable and the UEQ score as the dependent variable. The second exploratory question (E2) was tested using an 8 (EMA protocols) × 5 (nutrition app use stage) between‐subjects ANOVA with the respective sub‐scales for active information processing, eating‐related cognitions, intentions and eating behaviour change as the respective dependent variables; since a moderating effect was tested, the report focuses on the interaction of the two independent variables. The third exploratory question (E3) was tested using a between‐subjects ANCOVA, with the ATI score as a covariate and the respective sub‐scales for active information processing, eating‐related cognitions, intentions and eating behaviour change as the dependent variables.

Across analyses, *p* = .05 was used as the inference criterion. Reliability of scales was calculated using internal consistency (Cronbach's alpha).

## RESULTS

### Reliability analysis

Items reflecting the AIP (Cronbach's *α* = .82), eating‐related cognitions (Cronbach's *α* = .83), intentions (Cronbach's *α* = .80), behaviour (Cronbach's *α* = .83) and the ATI scale (Cronbach's *α* = .74) showed good internal consistencies with values above .7 (Tavakol & Dennick, 2011).

### H1: EMA protocols with more components are perceived to be more complex (manipulation check)

In line with the initial assumption, a between‐subjects ANOVA (*n* = 365) revealed significant differences in perceived complexity between EMA protocols. *F*(7, 365) = 16.82, *p* < .001, $\eta_{p}^{2}$ = .24. More specifically, EMA protocols that contained more tracking features were perceived to be more complex (perceived complexity – rated by the participants) (see Table 3 for means and standard deviations). Protocol 3, which included only the photo feature, was rated as significantly less complex than all other protocols (*p*s ≤ .030), except protocol 2, which included only the classification feature. Despite also only including one tracking feature (text‐based description), protocol 1 was perceived as significantly more complex than protocols 2 and 3 (*p*s < .001), but less complex than protocols 4 to 7 which contained 2–3 tracking features (*p*s ≤ .035); however, there was no significant difference between protocols 1 and 8, which included four tracking features. Protocol 7, which contained three features, was rated as significantly more complex than all other protocols (*p*s ≤ .005). Therefore H1 was largely supported. A list of all paired comparisons can be found in the online supplement.

**TABLE 3:** Complexity rating, active information processing ratings, anticipated changes to eating‐related cognitions, intentions and behaviours, and usability ratings (UEQ) for the eight EMA protocols.

EMA protocol	*N*	Complexity		AIP		Cognitions		Intentions		Behaviours		UEQ	
		*M*	*SD*	*M*	*SD*	*M*	*SD*	*M*	*SD*	*M*	*SD*	*M*	*SD*
1	51	2.99	.69	3.45	.85	3.80	.61	3.29	.81	3.10	.81	3.01	.84
2	56	2.30	.68	3.36	.73	3.76	.58	3.24	.69	3.21	.77	3.29	.64
3	53	2.25	.75	3.00	.92	3.73	.77	3.17	.69	3.03	.76	3.50	.52
4	42	2.69	.61	3.62	.63	3.91	.59	3.17	.72	3.10	.79	3.19	.67
5	36	2.66	.73	3.40	.84	3.95	.73	3.28	.72	3.16	.66	3.40	.74
6	42	2.56	.74	3.39	.81	3.80	.79	3.24	.82	3.05	.78	3.49	.75
7	43	3.40	.64	3.83	.55	3.99	.63	3.19	.88	3.15	.89	2.94	.67
8	50	3.19	.70	3.70	.55	4.00	.66	3.21	.77	3.18	.79	3.23	.70

### H2: The more complex the assessment tool, the more active information processing is required

A between‐subjects ANOVA (*n* = 373) revealed differences in AIP between EMA protocols, *F*(7, 373) = 5.63, *p* < .001, $\eta_{p}^{2}$ = .10. As expected, EMA protocols that were perceived to be more complex were also perceived to induce more active information processing, such as EMA protocol 7 (*M* = 3.83, *SD* = .55), compared to less complex EMA protocols, such as EMA protocol 3, which was rated as inducing significantly less AIP than all other protocols (*M* = 3.00, *SD* = .92; *p*s ≤ .012; see Table 3 for means and standard deviations of all groups and the online supplement for the paired comparisons). H2 was thus largely supported.

### H3: The more complex the assessment tool, the stronger its potential impact on participants' eating‐related cognitions, intentions and behaviour change

Between‐subjects ANOVAs (*n* = 373) indicate no significant differences between EMA protocols regarding perceived impact on eating‐related cognitions, intentions and behaviour (cognitions: *F*(7, 373) = 1.26, *p* = .270, $\eta_{p}^{2}$ = .02; intentions: *F*(7, 373) = .16, *p* = .993, $\eta_{p}^{2}$ = .01; behaviour: *F*(7, 373) = .33, *p* = .942, $\eta_{p}^{2}$ = .01; see Table 3 for means and standard deviations for all groups). H3 was thus not supported.

### E1: Do the assessment tools differ in usability?

A between‐subjects ANOVA (*n* = 373) revealed significant differences in usability between the different EMA protocols, *F*(7, 1) = 4.14, *p* < .001, $\eta_{p}^{2}$ = .07 (see Table 3 for means and standard deviations for all groups). The mock‐ups that were perceived to be less complex, such as protocol 3 (*M* = 3.50, *SD* = .52), were also perceived as more user‐friendly compared to protocols perceived as more complex, such as protocol 8 (*M* = 3.23, *SD* = .67). All paired comparisons are listed in the online supplement.

### E2: Does previous experience with nutrition apps influence participants' ratings of the hypothetical assessment tools?

Two‐way between‐subjects ANOVAs (*n* = 361) indicated that differences between EMA protocols in AIP (*F*(28, 333) = 1.30, *p* = .144, $\eta_{p}^{2}$ = .10), eating‐related cognitions (*F*(28, 333) = .58, *p* = .960, $\eta_{p}^{2}$ = .05), intentions (*F*(28, 333) = 1.25, *p* = .183, $\eta_{p}^{2}$ = .10) or behaviour (*F*(28, 333) = 1.15, *p* = .281, $\eta_{p}^{2}$ = .09) did not vary by participants' prior experience with using nutrition apps.

### E3: Does affinity to technology influence participants' ratings of the hypothetical assessment tools?

One‐way between‐subjects ANCOVAs (*n* = 364), using the ATI score as a covariate, indicated a significant main effect of EMA protocol AIP (*F*(1, 364) = 6.01, *p* = .015, $\eta_{p}^{2}$ = .02). Protocol 3 was rated to induce significantly less AIP compared to protocols 1, 5, 7 and 8 *p*s ≤ .046; means and standard deviations and p‐values for all paired comparisons are listed in the online supplement. The remaining paired comparisons did not reach statistical significance. However, there were no significant differences regarding eating‐related cognitions (*F*(1, 364) = 2.83, *p* = .094, $\eta_{p}^{2}$ = .01), intentions (*F*(1, 364) = 1.79, *p* = .182, $\eta_{p}^{2}$ = .01) and behaviour (*F*(1, 364) = 3.54, *p* = .061, $\eta_{p}^{2}$ = .01) when the ATI score was added as a covariate.

## DISCUSSION

A diverse range of smartphone‐based dietary assessment tools has been used in the literature (König, van Emmenis, et al., 2022), yet the potential effects of this variation on the comparability of findings across studies have remained mostly unexplored. This study compared the perceived complexity of different smartphone‐based dietary assessment tools and tested for potential differences in AIP and anticipated impact on eating‐related cognitions, intentions and behaviours. Results indicated that EMA protocols that use more dietary tracking features were indeed perceived to be more complex and less usable, and to induce more AIP in comparison to EMA protocols with fewer dietary tracking features. At the same time, however, the complexity of the EMA protocols was not perceived to differentially influence eating‐related cognitions, intentions and behaviours. Finally, previous experience with nutrition apps did not seem to have an impact on any of the investigated aspects of eating behaviour. Results were also mostly unchanged when ATI was included as a covariate.

The results of this study confirmed our notion that more assessment features increase the perceived complexity of a smartphone‐based dietary assessment tool; this is in line with previous research that has shown that the number of features or components in a task can influence participants' perception of its complexity (Goldschmidt et al., 2014; Heron & Smyth, 2010). Researchers might want to include more assessment features to gather more detailed information, especially when studying complex behaviours such as eating; yet our findings highlight the risk of both increasing perceived complexity and decreasing perceived usability, which might lead to underreporting, especially of snacks (Ziesemer et al., 2020), and dropout (König et al., 2021) and so reduce data quality. However, it is important to question whether recording tools indeed differentially influence compliance and dropout in EMA studies as the method is generally considered burdensome due to the number of assessments and disruptions to daily routines (Smyth et al., 2021). This assumption thus needs to be tested in future research to derive recommendations for researchers intending to use dietary EMA. Generally, however, it is advisable to carefully select the number and type of recording features carefully so as not to overburden participants, and to pay attention to usability, especially when using food databases (König et al., 2021).

Prior research indicates that the use of food databases and requiring participants to estimate portion sizes may be more burdensome than taking pictures of the food, which translates to willingness to use the tool (Höchsmann et al., 2021). Also in the present study, EMA protocols involving food photos were seen as less complex and more usable. It could thus be recommended to use photo‐based recordings instead of relying on food databases to increase compliance, although this has important implications regarding quantifying the recorded food intake (see König, van Emmenis, et al., 2022, for a discussion). It is important to note that the present study investigated dietary EMA in the context of assessing – not deliberately changing – a behaviour to describe it. In this context, changes are often undesired (c.f., measurement reactivity). However, self‐monitoring of (dietary) behaviour is also a frequently used behaviour change technique, which is often associated with greater intervention success (Michie et al., 2009). Thus, for the context of interventions, the present study provides different recommendations. Indeed, in line with Turner‐McGrievy et al. (2021), it could be suggested that if behaviour change is the goal, the burden associated with recording might actually produce stronger intervention effects and is therefore desired.

In line with H2, we also found that the complexity of the different EMA protocols was related to the perceived level of AIP they required. For instance, the EMA protocols that used a combination of tracking features (i.e., food database, detailed description and portion size assessment) were perceived to require more AIP in comparison to, for example, the EMA protocol that used only photo‐based assessment. This aligns with the concept of cognitive load, where tasks perceived as more complex or demanding require higher levels of cognitive effort (Davis, 1989). Moreover, in more complex dietary‐intake smartphone tracking apps, the interaction time with the tool is longer compared to less complex apps. Considering that participants in EMA studies typically track their behaviour repeatedly, this leads to an even larger amount of time spent actively processing diet‐related information as the study goes on.

Based on Control Theory (Carver & Scheier, 1982), it could be expected that these opportunities to reflect and detect discrepancies between the current and desired behaviour should induce changes in behaviour. Participants indeed indicated for all eight protocols that they might expect slight changes. This is also in line with prior qualitative work suggesting that EMA study participants feel that their behaviour may have changed due to taking part in a study (Eisele et al., 2023). However, contrary to our expectation, the protocols did not differ in this regard. Exploratory analyses also indicate that the ratings were not influenced by prior experience; participants who had used nutrition apps before did not expect the potential influences of the EMA protocols to be different from those of participants without prior experience. Still, it is important to note that this study was based on a hypothetical scenario, since participants only reflected on the mock‐ups and did not have the opportunity to use them. The protocols would need to be implemented and used to collect data in real life to test whether there are indeed no differences between the protocols, or whether these changes occur outside the individuals' awareness (Eisele et al., 2023). Shedding more light on the issue of changes in behaviour due to being measured in research, that is measurement reactivity (French & Sutton, 2010), is especially important for dietary EMA, since research in this domain is currently lacking (König, Allmeta, et al., 2022).

The study has several strengths, including that it was preregistered, and materials and data are shared on the OSF. The sample size was determined a priori and relatively large, yet due to a lack of prior research the effect size estimate was based on the mean effect size for psychological studies (Open Science Collaboration, 2015). The procedure was highly standardized, with all EMA protocols using the same design and consisting of the same basic features, so differences between protocols can indeed be attributed to the combination of features and not, for instance, the visual design (Valenčič et al., 2023). However, several limitations need to be noted. First, and most importantly, the study was hypothetical and relied on self‐reported expected changes. Since several studies indicated overreporting of desirable and underreporting of undesirable behaviours (Paulhus, 1991; Stone, 2007), the results need to be replicated in real‐life contexts; this would require programming apps that are identical in layout but differ in the type and number of implemented features. Unfortunately, given that the present study was conducted during the height of the Covid‐19 pandemic, this was not possible at the time. Still, self‐reported information also provides valuable insights, especially when later contrasted with objectively assessed data, since there is an ongoing debate in the literature as to when individuals notice changes in their behaviour (see Szymczak et al., 2020), and whether study participants are aware of changes to their behaviour due to the study context (Eisele et al., 2023). Furthermore, most constructs were assessed with items newly developed for this project, that require further validation if used again. Second, even though aspects such as affinity to technology, previous experience in using nutrition apps and the general usability (UEQ) of the EMA protocols were assessed, other aspects that might have been relevant that is, willingness to take part in a study using EMA protocols, were not included. Third, the study sample was mostly female, young and well educated; also former users of nutrition apps were likely overrepresented (König et al., 2018). We furthermore collected data internationally and managed recruitment from Germany; the fact that most participants were probably not English native speakers may have somewhat influenced their understanding of the mock‐ups, descriptions and items. However, the sample was relatively young and likely exposed to English regularly, both in school and through social and other media. The influence of the language on study outcomes is likely negligible. Still, generalizability to other samples thus remains to be tested. This sample composition, however, is typical for (dietary) EMA studies (see König, van Emmenis, et al., 2022, for an overview), so the conclusions drawn from this study still apply to many published studies. Finally, the sample was too small to detect small effects with sufficient power, thus small differences between EMA protocols regarding anticipated changes in eating‐related cognitions, intentions and behaviours might have been missed.

Smartphone‐based dietary EMA is becoming increasingly common in behavioural research, but apps are often developed by research teams themselves due to a lack of funds for commercial solutions or specific requirements of the research project. Aside from the influence on what information is collected, the type of tracking features may influence compliance with the study protocol, which in turn influences the completeness and informative value of the data. Researchers thus need to carefully select the appropriate EMA protocol for their study to balance the need for collected information with the need for high compliance.

## AUTHOR CONTRIBUTIONS

**Anila Allmeta:** Conceptualization; data curation; writing – original draft; formal analysis; investigation; methodology; project administration. **Stephen Sutton:** Conceptualization; methodology; writing – review and editing. **Laura M. König:** Conceptualization; methodology; writing – review and editing.

## CONFLICT OF INTEREST STATEMENT

The authors declare no competing interests.

## Supporting information


Appendix S1.

## Data Availability

Data and materials are available from the Open Science Framework: https://osf.io/4htx7/.

## References

[bjhp70057-bib-0001] Ambrosini, G. L. , Hurworth, M. , Giglia, R. , Trapp, G. , & Strauss, P. (2018). Feasibility of a commercial smartphone application for dietary assessment in epidemiological research and comparison with 24‐h dietary recalls. Nutrition Journal, 17(1), 5. 10.1186/s12937-018-0315-4 29316930 PMC5761106

[bjhp70057-bib-0002] Barta, W. D. , Tennen, H. , & Litt, M. D. (2012). Measurement reactivity in diary research. In M. R. Mehl & T. S. Conner (Eds.), Handbook of research methods for studying daily life: Chapter 6: Measurement reactivity in diary research. Guildford Press.

[bjhp70057-bib-0003] Béjar, L. M. , Reyes, Ó. A. , & García‐Perea, M. D. (2018). Electronic 12‐hour dietary recall (e‐12HR): Comparison of a Mobile phone app for dietary intake assessment with a food frequency questionnaire and four dietary records. JMIR mHealth and uHealth, 6(6), e10409. 10.2196/10409 29907555 PMC6026301

[bjhp70057-bib-0004] Biel, J.‐I. , Martin, N. , Labbe, D. , & Gatica‐Perez, D. (2018). Bites ‘n’ bits. Proceedings of the ACM on Interactive, Mobile, Wearable and Ubiquitous Technologies, 1(4), 1–33. 10.1145/3161161

[bjhp70057-bib-0005] Carver, C. S. , & Scheier, M. F. (1982). Control theory: A useful conceptual framework for personality–social, clinical, and health psychology. Psychological Bulletin, 92(1), 111–135. 10.1037/0033-2909.92.1.111 7134324

[bjhp70057-bib-0006] Clemes, S. A. , & Parker, R. A. (2009). Increasing our understanding of reactivity to pedometers in adults. Medicine & Science in Sports & Exercise, 41(3), 674–680. 10.1249/mss.0b013e31818cae32 19204581

[bjhp70057-bib-0007] Cohen, J. (1992). A power primer. Psychological Bulletin, 112(1), 155–159. 10.1037/0033-2909.112.1.155 19565683

[bjhp70057-bib-0008] Davis, F. D. (1989). Perceived usefulness, perceived ease of use, and user acceptance of information technology. MIS Quarterly, 13(3), 319. 10.2307/249008

[bjhp70057-bib-0009] Eisele, G. , Vachon, H. , Lafit, G. , Tuyaerts, D. , Houben, M. , Kuppens, P. , Myin‐Germeys, I. , & Viechtbauer, W. (2023). A mixed‐method investigation into measurement reactivity to the experience sampling method: The role of sampling protocol and individual characteristics. Psychological Assessment, 35(1), 68–81. 10.1037/pas0001177 36174163

[bjhp70057-bib-0010] Elliston, K. G. , Ferguson, S. G. , Schüz, N. , & Schüz, B. (2017). Situational cues and momentary food environment predict everyday eating behavior in adults with overweight and obesity. Health Psychology: Official Journal of the Division of Health Psychology, American Psychological Association, 36(4), 337–345. 10.1037/hea0000439 27669177

[bjhp70057-bib-0011] Faul, F. , Erdfelder, E. , Buchner, A. , & Lang, A.‐G. (2009). Statistical power analyses using G*Power 3.1: Tests for correlation and regression analyses. Behavior Research Methods, 41(4), 1149–1160. 10.3758/BRM.41.4.1149 19897823

[bjhp70057-bib-0012] Franke, T. , Attig, C. , & Wessel, D. (2019). A personal resource for technology interaction: Development and validation of the affinity for technology interaction (ATI) scale. International Journal of Human‐Computer Interaction, 35(6), 456–467. 10.1080/10447318.2018.1456150

[bjhp70057-bib-0013] French, D. P. , & Sutton, S. (2010). Reactivity of measurement in health psychology: How much of a problem is it? What can be done about it? British Journal of Health Psychology, 15(Pt 3), 453–468. 10.1348/135910710X492341 20205982

[bjhp70057-bib-0014] Goldschmidt, A. B. , Wonderlich, S. A. , Crosby, R. D. , Engel, S. G. , Lavender, J. M. , Peterson, C. B. , Crow, S. J. , Cao, L. , & Mitchell, J. E. (2014). Ecological Momentary Assessment of stressful events and negative affect in bulimia nervosa. Journal of Consulting and Clinical Psychology, 82(1), 30–39. 10.1037/a0034974 24219182 PMC3966065

[bjhp70057-bib-0015] Heilala, V. , Kelly, R. , Saarela, M. , Jääskelä, P. , & Kärkkäinen, T. (2022). The Finnish version of the affinity for technology interaction (ATI) scale: Psychometric properties and an examination of gender differences. International Journal of Human‐Computer Interaction, 39(4), 874–892. 10.1080/10447318.2022.2049142

[bjhp70057-bib-0016] Heron, K. E. , & Smyth, J. M. (2010). Ecological momentary interventions: Incorporating mobile technology into psychosocial and health behaviour treatments. British Journal of Health Psychology, 15(Pt 1), 1–39. 10.1348/135910709X466063 19646331 PMC2800172

[bjhp70057-bib-0017] Höchsmann, C. , Fearnbach, N. , Dorling, J. L. , Fazzino, T. L. , Myers, C. A. , Apolzan, J. W. , & Martin, C. K. (2021). Preference, expected burden, and willingness to use digital and traditional methods to assess food and alcohol intake. Nutrients, 13(10), 3340. 10.3390/nu13103340 34684341 PMC8539386

[bjhp70057-bib-0018] Jung, J. , Wellard‐Cole, L. , Cai, C. , Koprinska, I. , Yacef, K. , Allman‐Farinelli, M. , & Kay, J. (2020). Foundations for systematic evaluation and benchmarking of a mobile food logger in a large‐scale nutrition study. Proceedings of the ACM on Interactive, Mobile, Wearable and Ubiquitous Technologies, 4(2), 1–25. 10.1145/3397327 35846237

[bjhp70057-bib-0019] König, L. M. , Allmeta, A. , Christlein, N. , van Emmenis, M. , & Sutton, S. (2022). A systematic review and meta‐analysis of studies of reactivity to digital in‐the‐moment measurement of health behaviour. Health Psychology Review, 16(4), 551–575. 10.1080/17437199.2022.2047096 35264084

[bjhp70057-bib-0021] König, L. M. , & Renner, B. (2018). Colourful = healthy? Exploring meal colour variety and its relation to food consumption. Food Quality and Preference, 64, 66–71. 10.1016/j.foodqual.2017.10.011

[bjhp70057-bib-0022] König, L. M. , & Renner, B. (2019). Boosting healthy food choices by meal colour variety: Results from two experiments and a just‐in‐time ecological momentary intervention. BMC Public Health, 19(1), 975. 10.1186/s12889-019-7306-z 31331299 PMC6647103

[bjhp70057-bib-0020] König, L. M. , Attig, C. , Franke, T. , & Renner, B. (2021). Barriers to and facilitators for using nutrition apps: Systematic review and conceptual framework. JMIR mHealth and uHealth, 9(6), e20037. 10.2196/20037 34254938 PMC8409150

[bjhp70057-bib-0023] König, L. M. , Sproesser, G. , Schupp, H. T. , & Renner, B. (2018). Describing the process of adopting nutrition and fitness apps: Behavior stage model approach. JMIR mHealth and uHealth, 6(3), e55. 10.2196/mhealth.8261 29535078 PMC5871740

[bjhp70057-bib-0024] König, L. M. , van Emmenis, M. , Nurmi, J. , Kassavou, A. , & Sutton, S. (2022). Characteristics of smartphone‐based dietary assessment tools: A systematic review. Health Psychology Review, 16(4), 526–550. 10.1080/17437199.2021.2016066 34875978

[bjhp70057-bib-0025] Lakens, D. (2013). Calculating and reporting effect sizes to facilitate cumulative science: A practical primer for t‐tests and ANOVAs. Frontiers in Psychology, 4, 863. 10.3389/fpsyg.2013.00863 24324449 PMC3840331

[bjhp70057-bib-0026] Lezhnina, O. , & Kismihók, G. (2020). A multi‐method psychometric assessment of the affinity for technology interaction (ATI) scale. Computers in Human Behavior Reports, 1, 100004. 10.1016/j.chbr.2020.100004

[bjhp70057-bib-0027] Maher, J. P. , Arigo, D. , Baga, K. , Salvatore, G. M. , Pasko, K. , Hudgins, B. L. , & König, L. M. (2024). Measurement reactivity in Ecological Momentary Assessment studies of movement‐related behaviors. Journal for the Measurement of Physical Behaviour, 7(1). 10.1123/jmpb.2023-0035 PMC12922600 41726043

[bjhp70057-bib-0028] Michie, S. , Abraham, C. , Whittington, C. , McAteer, J. , & Gupta, S. (2009). Effective techniques in healthy eating and physical activity interventions: A meta‐regression. Health Psychology, 28(6), 690–701. 10.1037/a0016136 19916637

[bjhp70057-bib-0029] Miles, L. M. , Rodrigues, A. M. , Sniehotta, F. F. , & French, D. P. (2020). Asking questions changes health‐related behavior: An updated systematic review and meta‐analysis. Journal of Clinical Epidemiology, 123, 59–68. 10.1016/j.jclinepi.2020.03.014 32229251 PMC7308800

[bjhp70057-bib-0052] Natter, H. M. , & Berry, D. C. (2005). Effects of active information processing on the understanding of risk information. Applied Cognitive Psychology, 19(1), 123–135. 10.1002/acp.1068

[bjhp70057-bib-0030] Open Science Collaboration . (2015). Estimating the reproducibility of psychological science. Science (New York, N.Y.), 349(6251), aac4716. 10.1126/science.aac4716 26315443

[bjhp70057-bib-0031] Ottenstein, C. , & Werner, L. (2022). Compliance in ambulatory assessment studies: Investigating study and sample characteristics as predictors. Assessment, 29(8), 1765–1776. 10.1177/10731911211032718 34282659 PMC9597150

[bjhp70057-bib-0032] Paulhus, D. L. (1991). Measurement and control of response bias. In Measures of personality and social psychological attitudes (pp. 17–59). Elsevier. 10.1016/B978-0-12-590241-0.50006-X

[bjhp70057-bib-0033] Pendergast, F. J. , Livingstone, K. M. , Worsley, A. , & McNaughton, S. A. (2016). Correlates of meal skipping in young adults: A systematic review. International Journal of Behavioral Nutrition and Physical Activity, 13(1), 125. 10.1186/s12966-016-0451-1 27905981 PMC5133750

[bjhp70057-bib-0034] Perski, O. , Keller, J. , Kale, D. , Asare, B. Y.‐A. , Schneider, V. , Powell, D. , Naughton, F. , ten Hoor, G. , Verboon, P. , & Kwasnicka, D. (2022). Understanding health behaviours in context: A systematic review and meta‐analysis of Ecological Momentary Assessment studies of five key health behaviours. Health Psychology Review, 16(4), 576–601. 10.1080/17437199.2022.2112258 35975950 PMC9704370

[bjhp70057-bib-0035] Pew Research Center . (2020). Mobile Fact Sheet . https://www.pewresearch.org/internet/fact‐sheet/mobile/

[bjhp70057-bib-0036] Reichenberger, J. , Smyth, J. M. , Kuppens, P. , & Blechert, J. (2019). “I will fast … Tomorrow”: Intentions to restrict eating and actual restriction in daily life and their person‐level predictors. Appetite, 140, 10–18. 10.1016/j.appet.2019.04.019 31039371

[bjhp70057-bib-0051] Scholz, U. (2019). It's time to think about time in health psychology. Applied Psychology. Health and Well‐Being, 11(2), 173–186. 10.1111/aphw.12156 30972951

[bjhp70057-bib-0037] Schrepp, M. , Hinderks, A. , & Thomaschewski, J. (2017). Design and evaluation of a short version of the user experience questionnaire (UEQ‐S). International Journal of Interactive Multimedia and Artificial Intelligence, 4(6), 103. 10.9781/ijimai.2017.09.001

[bjhp70057-bib-0038] Seifert, A. , Schlomann, A. , Rietz, C. , & Schelling, H. R. (2017). The use of mobile devices for physical activity tracking in older adults' everyday life. DIGITAL HEALTH, 3, 2055207617740088. 10.1177/2055207617740088 29942617 PMC6001246

[bjhp70057-bib-0039] Sharp, D. B. , & Allman‐Farinelli, M. (2014). Feasibility and validity of mobile phones to assess dietary intake. Nutrition, 30(11–12), 1257–1266. 10.1016/j.nut.2014.02.020 24976425

[bjhp70057-bib-0050] Shiffman, S. , Stone, A. A. , & Hufford, M. R. (2008). Ecological momentary assessment. Annual Review of Clinical Psychology, 4(1), 1–32. 10.1146/annurev.clinpsy.3.022806.091415 18509902

[bjhp70057-bib-0040] Smyth, J. M. , Jones, D. R. , Wen, C. K. F. , Materia, F. T. , Schneider, S. , & Stone, A. (2021). Influence of Ecological Momentary Assessment study design features on reported willingness to participate and perceptions of potential research studies: An experimental study. BMJ Open, 11(7), e049154. 10.1136/bmjopen-2021-049154 PMC8327852 34330860

[bjhp70057-bib-0041] Stone, A. A. (2007). The science of real‐time data capture: Self‐reports in health research. Oxford University Press.

[bjhp70057-bib-0042] Szymczak, H. , Keller, L. , Debbeler, L. J. , Kollmann, J. , Lages, N. C. , Sproesser, G. , Gollwitzer, P. M. , Schupp, H. T. , & Renner, B. (2020). “I'm eating healthy now”: The relationship between perceived behavior change and diet. Food Quality and Preference, 89, 104142. 10.1016/j.foodqual.2020.104142

[bjhp70057-bib-0043] Tavakol, M. , & Dennick, R. (2011). Making sense of Cronbach's alpha. International Journal of Medical Education, 2, 53–55. 10.5116/ijme.4dfb.8dfd 28029643 PMC4205511

[bjhp70057-bib-0044] Turner‐McGrievy, G. M. , Yang, C.‐H. , Monroe, C. , Pellegrini, C. , & West, D. S. (2021). Is burden always bad? Emerging low‐burden approaches to Mobile dietary self‐monitoring and the role burden plays with engagement. Journal of Technology in Behavioral Science, 6(3), 447–455. 10.1007/s41347-021-00203-9

[bjhp70057-bib-0045] University of Lübeck . (2025). ATI Scale. https://ati‐scale.org/ (accessed September 28, 2025).

[bjhp70057-bib-0046] Valenčič, E. , Beckett, E. , Collins, C. E. , Koroušić Seljak, B. , & Bucher, T. (2023). Snacktrack‐an app‐based tool to assess the influence of digital and physical environments on snack choice. Nutrients, 15(2), 349. 10.3390/nu15020349 36678219 PMC9862135

[bjhp70057-bib-0047] Wansink, B. , & Sobal, J. (2006). Mindless eating. Environment and Behavior, 39(1), 106–123. 10.1177/0013916506295573

[bjhp70057-bib-0048] Wessel, D. , Attig, C. , & Franke, T. (2019). ATI‐S – An ultra‐short scale for assessing affinity for technology interaction in user studies. In F. Alt , A. Bulling , & T. Döring (Eds.), Proceedings of mensch und computer 2019 (pp. 147–154). ACM. 10.1145/3340764.3340766

[bjhp70057-bib-0049] Wrzus, C. , & Neubauer, A. B. (2023). Ecological Momentary Assessment: A meta‐analysis on designs, samples, and compliance across research fields. Assessment, 30(3), 825–846. 10.1177/10731911211067538 35016567 PMC9999286

[bjhp70057-bib-0053] Ziesemer, K. , König, L. M. , Boushey, C. J. , Villinger, K. , Wahl, D. R. , Butscher, S. , Müller, J. , Reiterer, H. , Schupp, H. T. , & Renner, B. (2020). Occurrence of and reasons for “missing events” in mobile dietary assessments: Results from three event‐based ecological momentary assessment studies. JMIR mHealth and uHealth, 8(10), e15430. 33052123 10.2196/15430 PMC7593856

