# Supplementary material for: The same, only different: Smartphone‐based dietary Ecological Momentary Assessment tools vary in complexity, usability and active information processing
Source: Br J Health Psychol. 2026 Jan 28;31(1):e70057. doi: 10.1111/bjhp.70057 (PMC12853076; doi:10.1111/bjhp.70057)
Supplement: Supplementary file 1 — Appendix S1. [file BJHP-31-0-s001.docx]

**Online supplementary material**

**Table S1.** Paired comparisons between EMA protocols for perceived complexity, active information processing (AIP), and usability, as measured by the User Experience Questionnaire (UEQ).

| EMA protocol | Compared to EMA protocol | Complexity | AIP | UEQ |
| --- | --- | --- | --- | --- |
| 1 | 2 | <.001 | 1.00 | .037 |
|  | 3 | <.001 | .002 | <.001 |
|  | 4 | .035 | 1.00 | 1.00 |
|  | 5 | .027 | 1.00 | .010 |
|  | 6 | .003 | 1.00 | <.001 |
|  | 7 | .005 | .016 | 1.00 |
|  | 8 | .149 | 1.00 | 1.00 |
| 2 | 3 | .680 | .012 | 1.00 |
|  | 4 | .008 | 1.00 | .011 |
|  | 5 | .019 | 1.00 | 1.00 |
|  | 6 | .071 | 1.00 | 1.00 |
|  | 7 | <.001 | .002 | .309 |
|  | 8 | <.001 | .018 | 1.00 |
| 3 | 4 | .003 | <.001 | .034 |
|  | 5 | .007 | .012 | 1.00 |
|  | 6 | .030 | .011 | 1.00 |
|  | 7 | <.001 | <.001 | <.001 |
|  | 8 | <.001 | .012 | .048 |
| 4 | 5 | .854 | 1.00 | 1.00 |
|  | 6 | .418 | 1.00 | .045 |
|  | 7 | <.001 | 1.00 | 1.00 |
|  | 8 | <.001 | 1.00 | 1.00 |
| 5 | 6 | .552 | 1.00 | 1.00 |
|  | 7 | <.001 | .013 | .003 |
|  | 8 | <.001 | .970 | 1.00 |
| 6 | 7 | <.001 | .008 | <.001 |
|  | 8 | <.001 | .046 | 1.00 |
| 7 | 8 | .163 | 1.00 | .045 |

**Table S2.** Means and standard deviations for the ANCOVA testing group differences in AIP between EMA protocols controlling for affinity to technology (ATI), and p-values for all paired comparisons.

| EMA protocol | M | SD | P-values for paired comparisons | | | | | | |
| --- | --- | --- | --- | --- | --- | --- | --- | --- | --- |
|  |  |  | 2 | 3 | 4 | 5 | 6 | 7 | 8 |
| 1 | 3.45 | 0.85 | 1.00 | .046 | 1.00 | 1.00 | 1.00 | .521 | 1.00 |
| 2 | 3.36 | 0.73 |  | .178 | 1.00 | 1.00 | 1.00 | .161 | .538 |
| 3 | 2.99 | 0.92 |  |  | .001 | .254 | .250 | < .001 | <.001 |
| 4 | 3.62 | 0.63 |  |  |  | 1.00 | 1.00 | 1.00 | 1.00 |
| 5 | 3.41 | 0.84 |  |  |  |  | 1.00 | .390 | 1.00 |
| 6 | 3.40 | 0.81 |  |  |  |  |  | .254 | .817 |
| 7 | 3.83 | 0.55 |  |  |  |  |  |  | 1.00 |
| 8 | 3.71 | 0.55 |  |  |  |  |  |  |  |
